# Wet feet: developing sulfur isotope provenance methods to identify wetland inhabitants

**DOI:** 10.1098/rsos.230391

**Published:** 2023-10-11

**Authors:** Angela L. Lamb, Carolyn A. Chenery, Richard Madgwick, Jane A. Evans

**Affiliations:** ^1^ National Environmental Isotope Facility, British Geological Survey, Keyworth, Nottingham NG12 5GG, UK; ^2^ School of History, Archaeology and Religion, Cardiff University, Cardiff CF10 3EU, UK

**Keywords:** sulfur, isotopes, provenance, wetland, archaeological, biosphere

## Abstract

The stable isotopes of sulfur provide a distinctive signature for marine proximity and interaction. Exploring coastal proximity has been the principal application of sulfur isotopes in archaeology and palaeoecology, but this deals only with high (greater than 14‰) isotope values, meaning little interpretation has been gained from lower values. Progress has been hindered by issues with biosphere mapping. Air pollution can impact modern landscapes, significantly lowering sulfur isotope baselines, leading to the assumption that modern vegetation-based sulfur maps are not reliable. This research explores the potential of previously undiagnostic low, and often, negative sulfur isotope values for identifying wetland dwellers. Impervious clays that support wetlands are distinctive ecosystems and this study tests the hypothesis that they will produce low isotope values owing to both the underlying substrate and to redox conditions. Primary mapping of targeted areas using modern plants highlights zones with natural negative sulfur values and demonstrates that this constitutes a distinctive wetland signature. Analysis of modern and archaeological fauna demonstrates that these distinctive isotope compositions are transferred into the food chain. These findings propel the interpretative potential of sulfur isotopes forward and add to the growing knowledge to provide means for identifying archaeological humans and animals raised in wetlands.

## 1. Introduction

Identifying migrants and human/animal movement has long been an important pursuit in archaeology. Isotope analysis provides direct data for this and is helpful for identifying non-local individuals and patterns of migration. Strontium, relating to geology, and oxygen, to climate, are the most commonly applied isotope systems, but even when used together can be ambiguous in assigning origins. Sulfur isotopes in proteins preserved in archaeological material have predominantly been used to distinguish between marine and terrestrial diets (see [1] for review), being especially useful when the dietary interpretation of nitrogen isotopes is complicated by aridity or nutritional stress. There is only a minor offset between *δ*^34^S in diet and the consumer (0–0.5‰, [2]) and little fractionation through trophic levels. By contrast there is a large range in *δ*^34^S from terrestrial to marine ecosystems. Marine primary producers have *δ*^34^S values between +17 to +21‰, reflecting marine sulfates with the effects of sea-spray and marine precipitation extending this effect to coastal ecosystems and thus resulting in much higher *δ*^34^S soil and vegetation values within at least 20 km of the coast [3]. By contrast, terrestrial organisms have much lower, more variable, values reflecting the relative uptake of sulfate and sulfides (reflecting underlying soil processes; [4]), hydrological processes and atmospheric SO_2_ (typically –7 to +8‰; [5]). In freshwater environments such as marshes, and other waterlogged environments, soil microbial activity leads to sulfide production and extremely low *δ*^34^S values, typically −25‰ to –30‰ and occasionally as low as –62‰ [5–7]. Most plants will uptake sulfur in sulfate form, but some plants are adapted to sulfide uptake [8] and in these circumstances the production of biogenic sulfide can create highly depleted biosphere ^34^S [9]. This process, however, can be highly variable across ecosystems, sites, plant species and even within individual plants [5] and thus is a complex biosphere indicator. Evidence that reduced sulfur could generate low *δ*^34^S values in non-tidal wetland ecosystems was first demonstrated in a prairie marsh in Manitoba, Canada [10]. This has been subsequently validated by Guiry *et al.* [11] who demonstrated that wetland fauna incorporate sulfide *δ*^34^S through the food chain and subsequently preserve a low *δ*^34^S value that maybe significantly lower than the local baseline and there are several examples of fauna taking up sulfur depleted in ^34^S resulting from elevated soil sulfide conditions [12]. In such environments, at the same time, *δ*^15^N can be increased owing to nitrification-denitrification processes [13]. This recent finding, along with work on sulfur isotopes in riverine sites illustrate the complexity of freshwater isotope ecology and the potential issues this raises in archaeological studies. Understanding is growing of the need to incorporate soil-hydrosphere processes in studies of current and historical wetland environments [11,13,14]. Differences in terrestrial and riverine sulfate sources have been successfully used to distinguish food sources [15], however others have found that riverine and terrestrial *δ*^34^S biospheres can overlap and therefore in some environments sulfur isotopes cannot be used to distinguish these relative food sources in isolation [16]. Further, bone recovered from riverbank sites can become contaminated with exogenous sulfur causing diagenetic alteration in *δ*^34^S values [17].

Thus, to date, sulfur isotopes have had most application differentiating between coastal and inland food resourcing [18] and animal management [19], distinguishing between the consumption of freshwater aquatic and terrestrial protein [15] and as part of multi-isotope strategies [20–24]. The inclusion of sulfur isotopes in multi-proxy studies has also lagged behind other isotope systems owing to the technical challenges of measuring sulfur isotope ratios in collagen owing to its low concentration and the resulting large sample necessary to overcome this. However, recent methodological advancements have allowed smaller amounts of organic material to be analysed along with the ability to simultaneously analyse carbon, nitrogen and sulfur [25]. With improvements in technical aspects, one enduring barrier to the application of non-coastal biosphere sulfur isotopes is the impact of modern pollution on biosphere values. Dietary *δ*^34^S will reflect the sulfate composition of the substrate of the primary organisms [7] and thus has the potential to identify ‘non-locals’ in a population if there is supporting baseline sulfate information (e.g. [18]).

### 1.1. Biosphere mapping

Beyond its use as a coastal/marine indicator, issues with biosphere mapping have been a major barrier to the uptake of sulfur isotope analysis in archaeology. Local sulfur biospheres can be highly complex and variable through time and space [1,15,26]. Industrial SO_2_ pollution has long been considered problematic for sulfur biosphere mapping [27]. A linear relationship has been demonstrated between UK SO_2_ emissions and both herbage sulfur concentration and *δ*^34^S with increases in emissions coupled with lower *δ*^34^S values and higher sulfur concentrations [28,29]. Other impacts include the addition of agricultural fertilizers to the biosphere [30] and mining-related hydrological pollution [31]. These examples have led to the belief that modern vegetation-based sulfur baseline maps are not valid for reconstructing past mobility (see review [1]). Comparing modern and archaeological collagen samples from seven UK archaeological sites, Richards *et al.* [27] illustrated that all the modern faunal collagen from inland central and southern England had negative sulfur isotope values and were inconsistent with the archaeological material. Only the material from coastal north Wales and inland northern Scotland were consistent in isotope value with the archaeological material. This led Richards *et al*. [27] to suggest that the disconnect between modern and archaeological fauna was owing to the uneven impact of industrial pollution in the UK and thus it was better to use site faunal material as a sulfur baseline rather than modern plant/animal material. This premise is widely held in the literature, although there are studies which have explored the connection and have concluded that in some circumstances the modern biosphere is representative of the baseline values and that it is imperative to understand the environmental context of each site [32]. Additionally, following UK legislation in the 1950s and 1960s, the Department for Environment, Food and Rural Affairs has recorded a dramatic decline in SO_2_ emissions (from approx. 3500 kt in 1992 to less than 200 kt in 2017 [33]) with this reduction evident in lower biosphere pollutant sulfur loading [34]. Negative sulfur isotope values form a small percentage of human and animal data found in the UK. Few studies have produced large datasets in Britain. Among faunal studies, only one negative value from a total of 123 measurements (mean +11.7‰, 1 s.d. 4.0‰) was produced in a study on Neolithic pigs from southern Britain [22] and none (mean +15.2‰, 1 s.d. 1.1‰) were produced in a study of 29 Iron Age domesticates from Northern Ireland [21]. In a study of 22 fallow deer from across England (mean +10.0‰, 1 s.d. 6.8‰), only two produced negative values (–2.5‰ and –11.3‰), both from Goltho, a site overlying Jurassic mudstone in Lincolnshire ([19], unpublished Dama International project data 2013). Four studies have produced a higher proportion of negative values. Research on fauna from Late Bronze Age/Early Iron Age middens in Wiltshire and the Thames Valley, southern Britain, produced 59 negative values from 220 samples (mean +4.7‰, 1 s.d. 8.7‰) [35]. A study on the Bronze Age barrows of Gayhurst and Irthlingborough, in a region with Jurassic mudstone lithology, produced 11 negative values from 21 samples (mean –0.3‰, 1 s.d. 3.1‰, [36]). Nehlich and colleagues [32] study on Roman Oxfordshire, in an area dominated by Jurassic mudstone, produced 10 negative values from 11 samples (mean –5.5‰, 1 s.d. 5.1‰). Finally, a study of Early Bronze Age people from across Great Britain [37] gave an average *δ*^34^S of +11.7‰ ± 4.6 (*n* = 488, 1 s.d.) of which 14 samples have sulfur isotope values below zero and the two lowest values are from samples found in Lincolnshire on Jurassic clays.

These aforementioned studies along with recent mapping of sulfur in modern plants shows a strong correlation between negative sulfur isotope values and particular rock types, notably Jurassic mudstones, in inland southern England [38]. These impervious clays widely result in wetland environments and are prone to waterlogging and thus sulfate reduction [39]. The resulting disseminated sulfides in bedrock, soils and groundwater produce significantly lower biosphere sulfur isotope values, which may be assimilated by some plants that have adapted to sulfides [8]. The impact of impervious lithology on the UK sulfur biosphere is demonstrated by an insect-based isoscape of the UK and Ireland [40]. The sulfur isotope ratios of almost 300 moths from 93 locations were measured by Newton [40] and mapped. The mean *δ*^34^S value was 4.4‰ but ranged from –18.1‰ to +15.1‰ with the very lowest values clustered in the region of southern England underlain by Jurassic mudstones and ironstones. Few studies have explored the relationship between lithology and sulfur isotopes, with a notable exception in Northern Ireland, where a clear relationship was evident between parent rock/soil type and *δ*^34^S values, with the lowest values found on mudstones, in particular those with gley soils and thus those with sulfate reduction processes likely [41].

There has been some recent work that has begun to develop primary methods of identifying past humans and animals raised in wetland environments, both in freshwater [11] and coastal/marine settings [13]. Refining and developing these methods are vital to the archaeologist's toolkit, as wetlands represent exceptionally important and vibrant areas of occupation and exploitation in the past. Wetlands provide fertile zones for animal husbandry and are often in areas where freshwater fishes are in plentiful supply. Therefore, it should be no surprise that many high-profile prehistoric sites are founded on wetlands, including Glastonbury Lake Village, Must Farm, Flag Fen, Star Carr and the Neolithic lake dwellings of circum-alpine central Europe. The absence of a method to identify origins in these areas, especially as the Jurassic mudstone provides a very undiagnostic ^87^Sr/^86^Sr composition of around 0.709, means their role in past networks often remains poorly understood. Thus, the central aim of this study is to examine if sulfur isotopes can provide a method for identifying humans and animals that lived in wetland areas. Through primary analysis of modern plants, this study explores whether this relationship is consistent across similar lithologies and the extent to which negative sulfur isotope values are diagnostic for this geological type. In addition, modern and archaeological fauna are analysed to establish whether these distinctive sulfur isotope values are transferred into the food chain and preserved.

Our hypothesis is (i) that certain rock formations in England have relatively low (often negative) *δ*^34^S values that are transmitted into the soil and biosphere; (ii) these clays and mudrocks tend to be impervious and readily form wetlands such as the Somerset Levels, which further support anaerobic conditions and low *δ*^34^S values; (iii) that the low and negative *δ*^34^S values generated by these impervious anaerobically formed clays are transmitted into the flora and fauna of such wetland and provide a signature for these environments; and (iv) that these values are natural and not substantially impacted by modern pollution.

## 2. Material and methods

To characterize the modern sulfur biosphere of typical wetland lithologies, we collected and analysed modern plant samples from along the Jurassic outcrops of southern England, targeting archaeologically important areas of the Somerset Levels and the Cambridgeshire Fens (58 plant samples).

To understand if these plant signals were transferred to the fauna, we analysed sulfur isotopes in bone collagen extracted from locally raised farm animals in these areas (nine individuals). The samples were obtained from farm shops with known animal grazing provenance information. To test if this relationship held in archaeological samples, we analysed sulfur isotopes in bone collagen extracted from faunal material from archaeological sites from both regions to compare with modern data and as a baseline for expected faunal values in the respective regions (65 individual faunal samples). The archaeological material was obtained from three sites in the Somerset Levels: Beckery Chapel, Burtle Priory and Muchelney (sampling permission from South West Heritage Trust) and three sites in the Cambridgeshire Fens: Langtoft, Eye and Over (sampling permission from Cambridge Archaeological Unit).

### 2.1. Plant samples

**Figure 1.**
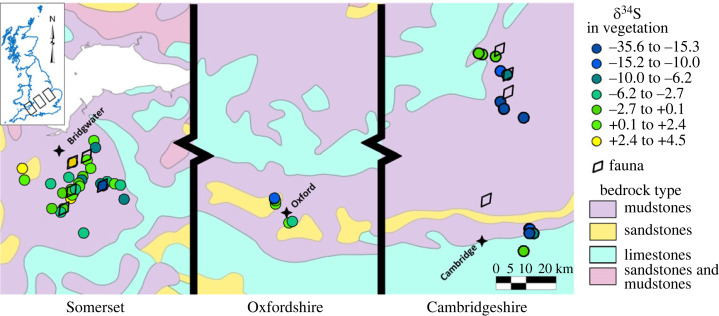
Location and sulfur isotope composition of plant samples and location of modern faunal samples. Contains Ordnance Survey data © Crown copyright and database right 2023.

Fifty-eight plant samples were collected from Oxfordshire, the Somerset Levels and the Cambridgeshire Fens (figure 1) with the aim of providing transects across mudstone lithologies in areas close to (or in similar habitats to) the archaeological sites and in the case of Oxfordshire, in an area that has previously produced low sulfur isotope values in archaeological fauna [32]. A total of 90% of the plant samples are from mudstones in areas subject to groundwater and/or seasonal flooding, with exact locations in table 1.

**Table 1.** Location and sulfur isotope composition of modern plants collected from wetland regions in southern England.

sample	*δ*^34^S_VCDT_ ‰	%S	latitude	longitude	elevation (m)	region	bedrock/age-Epoch
BAP-01	−0.8	0.1	52.65519	−0.41179	27.99	Lincolnshire	mudstone/ Mid-Late Jurassic
BAP-02	0.9	0.1	52.658	−0.39441	15.88	Lincolnshire	mudstone/ Mid Jurassic
BAP-03	−1.8	0.1	52.67574	−0.33696	8.60	Cambridgeshire	mudstone/ Mid-Late Jurassic
BAP-04	−14.8	0.1	52.64885	−0.27648	3.80	Cambridgeshire	mudstone/ Mid-Late Jurassic
BAP-06	−6.9	0.1	52.65072	−0.24177	4.26	Cambridgeshire	mudstone/ Mid-Late Jurassic
BAP-10	−18.1	0.1	52.57442	−0.19288	2.01	Cambridgeshire	mudstone/ Mid-Late Jurassic
BAP-11	−24.6	0.2	52.56342	−0.15931	−4.45	Cambridgeshire	mudstone/ Mid-Late Jurassic
BAP-12	−31.6	0.2	52.56216	−0.1591	−10.35	Cambridgeshire	mudstone/ Mid-Late Jurassic
BAP-13	−18.4	0.4	52.57451	−0.05671	2.99	Cambridgeshire	mudstone/ Mid-Late Jurassic
BAP-14	−20.2	0.1	52.57451	−0.05671	2.99	Cambridgeshire	mudstone/ Mid-Late Jurassic
BAP-15	−14.4	0.2	52.57451	−0.05671	2.99	Cambridgeshire	mudstone/ Mid-Late Jurassic
SL-01	0.2	0.1	51.68043	−1.25498	59.84	Oxfordshire	mudstone/ Late Jurassic
SL-02	−5.8	0.1	51.6893	−1.24486	63.74	Oxfordshire	mudstone/ Late Jurassic
SL-03	1.0	0.1	51.71004	−1.36548	87.79	Oxfordshire	sandstone, siltstone, mudstone/ Late Jurassic
SL-04	−12.6	0.1	51.71484	−1.37753	63.06	Oxfordshire	mudstone/ Mid-Late Jurassic
SL-05	−7.5	0.1	51.71278	−1.37567	66.43	Oxfordshire	mudstone/ Mid-Late Jurassic
SL-06	1.1	0.1	51.70287	−1.35661	90.94	Oxfordshire	sandstone/ Late Jurassic
SL-07.1	−9.8	0.2	51.14961	−2.61441	28.64	Somerset	interbedded mudstone and limestone/ Late Triassic-Early Jurassic
SL-08	−4.7	0.2	51.16193	−2.72634	4.55	Somerset	interbedded mudstone and limestone/ Late Triassic-Early Jurassic
SL-09	0.0	0.1	51.13153	−2.83216	78.53	Somerset	interbedded mudstone and limestone/ Late Jurassic
SL-10	−7.4	0.1	51.12106	−2.82632	7.25	Somerset	mudstone and halite-stone/ Early-Late Triassic
SL-11	0.1	0.1	51.10904	−2.82754	6.56	Somerset	mudstone and halite-stone/ Early-Late Triassic
SL-12	−4.0	0.3	51.10296	−2.81714	4.15	Somerset	mudstone and halite-stone/ Early-Late Triassic
SL-13	1.0	0.3	51.10272	−2.81632	4.16	Somerset	mudstone and halite-stone/ Early-Late Triassic
SL-15	−3.0	0.3	51.08827	−2.82362	4.64	Somerset	mudstone and halite-stone/ Early-Late Triassic
SL-17	−1.3	0.1	51.08043	−2.83905	101.42	Somerset	interbedded mudstone and limestone/ Late Triassic-Early Jurassic
SL-18	1.8	0.2	51.07596	−2.82217	96.45	Somerset	interbedded mudstone and limestone/ Late Triassic
SL-19	0.4	0.1	51.06928	−2.82024	82.52	Somerset	interbedded mudstone and limestone/ Late Triassic-Early Jurassic
SL-20	2.6	0.1	51.05867	−2.81663	39.82	Somerset	interbedded mudstone and limestone/ Late Triassic
SL-21	1.4	0.2	51.01972	−3.04134	8.45	Somerset	mudstone and halite-stone/ Early-Late Triassic
SL-22	3.8	0.2	51.04416	−3.08143	31.16	Somerset	mudstone and halite-stone/ Early-Late Triassic
SL-23	−3.7	0.3	51.05796	−2.93324	5.49	Somerset	mudstone and halite-stone/ Early-Late Triassic
SL-24	4.7	0.2	51.14668	−2.91081	9.77	Somerset	mudstone and halite-stone/ Early-Late Triassic
SL-26	−19.7	0.4	51.14465	−2.72994	6.80	Somerset	interbedded mudstone and limestone/ Late Triassic-Early Jurassic
SL-27	0.2	0.4	51.17023	−2.69176	4.87	Somerset	interbedded mudstone and limestone/ Late Triassic-Early Jurassic
SL-29	−3.2	0.2	51.17767	−2.66749	4.77	Somerset	interbedded mudstone and limestone/ Late Triassic
SL-30	2.5	0.1	51.16461	−2.82452	2.76	Somerset	interbedded mudstone and limestone/ Late Triassic-Early Jurassic
SL-31	2.2	0.1	51.16461	−2.82452	2.76	Somerset	interbedded mudstone and limestone/ Late Triassic-Early Jurassic
SL-32	1.7	0.2	51.16506	−2.82418	2.60	Somerset	interbedded mudstone and limestone/ Late Triassic-Early Jurassic
SL-33	−8.6	0.2	51.22161	−2.8648	10.11	Somerset	mudstone/ Early Jurassic
SL-34	1.7	0.3	51.23548	−2.89205	5.55	Somerset	mudstone/ Early Jurassic
SL-35	−3.8	0.2	50.99921	−2.67167	22.38	Somerset	interbedded mudstone and limestone/ Late Triassic-Early Jurassic
SL-36	−2.3	0.2	51.02062	−2.8147	11.53	Somerset	interbedded mudstone and limestone/ Late Triassic-Early Jurassic
SL-37	−5.3	0.3	51.00836	−2.81418	9.18	Somerset	interbedded mudstone and limestone/ Late Triassic-Early Jurassic
SL-38	−1.4	0.2	51.00157	−2.85743	25.19	Somerset	interbedded mudstone and limestone/ Late Triassic-Early Jurassic
SL-39	−0.1	0.2	51.05764	−2.86022	5.48	Somerset	mudstone and halite-stone/ Early-Late Triassic
SL-40	−4.1	0.3	51.10479	−2.8831	3.45	Somerset	mudstone and halite-stone/ Early-Late Triassic
SL-41	−7.7	0.4	51.14582	−2.74533	4.59	Somerset	interbedded mudstone and limestone/ Late Triassic-Early Jurassic
SL-42	1.7	0.4	51.17839	−2.86136	2.07	Somerset	interbedded mudstone and limestone/ Late Triassic-Early Jurassic
WIK-01	−1.6	0.2	52.25134	0.302132	25.91	Cambridgeshire	chalk/ Late Cretaceous
WIK-02	−1.2	0.2	52.25113	0.302239	23.12	Cambridgeshire	chalk/ Late Cretaceous
WIK-03	−4.0	0.5	52.30724	0.28533	1.68	Cambridgeshire	mudstone/ Early Cretaceous
WIK-04	−29.6	0.5	52.30462	0.277978	1.07	Cambridgeshire	mudstone/ Early Cretaceous
WIK-05	−28.8	0.3	52.30462	0.277978	1.07	Cambridgeshire	mudstone/ Early Cretaceous
WIK-06	−8.9	0.1	52.3108	0.295108	7.18	Cambridgeshire	mudstone/ Early Cretaceous
WIK-07	−14.7	0.2	52.31448	0.269491	1.69	Cambridgeshire	limestone/ Late Jurassic
WIK-08	−4.1	0.1	52.31501	0.265997	4.15	Cambridgeshire	limestone/ Late Jurassic
WIK-09	−2.3	0.0	52.31481	0.264651	5.58	Cambridgeshire	limestone/ Late Jurassic

### 2.2. Modern fauna

Modern faunal samples were targeted from grass-fed sheep and cattle from the Somerset Levels and Cambridgeshire Fens. Sourcing bones from animals of known life history and provenance proved challenging and consequently sample numbers are small. A total of seven cattle and two sheep samples were acquired from butchers and farm shops, where husbandry in local wetland environments for a large part of their lives could be assured and where diets were not supplemented from non-wetland sources. The modern faunal samples were collected in 2020. Details of the samples are presented in the electronic supplementary material, table S1.

### 2.3. Archaeological fauna

A total of 68 caprine and cattle samples were analysed from six sites on the Somerset Levels and Cambridgeshire Fens. Sample details are presented in the electronic supplementary material, table S2 and brief site descriptions are below.

Three sites were sampled from the Somerset Levels, with six cattle and six caprines (probably dominated by sheep) analysed from each site. The first, Muchelney is located *ca* 2 km south of the town of Langport in the heart of the Somerset Levels. The site overlies interbedded mudstone (Charmouth formation) and limestone (Langport Member Blue Lias formation). The area was an island in antiquity prior to drainage and was the site of a major abbey in the medieval period. Faunal remains were recovered from excavations to the east of the abbey [42] and date to the early and middle Roman period. The faunal assemblage is typical of a Roman rural settlement in southern Britain [43].

Burtle Priory, located just north of the Polden Hills, was an Augustinian priory in the medieval period. It became a priory cell of Glastonbury Abbey in the thirteenth century and was dissolved in 1536. Faunal remains are from community excavations by Brunning [44] and span the high and late medieval period. The site is founded on peat that overlies interbedded mudstone (Charmouth formation) and limestone (Langport Member Blue Lias formation). The faunal assemblage is dominated by the three main domesticates but has fewer pigs than is typical of contemporaneous monastic assemblages, perhaps owing to the Levels, with little woodland, being less suited to pig husbandry [45].

Beckery Chapel, also known as St Bridget's chapel, was situated on the highest part of Beckery Hill, an island in antiquity that is now on the western edge of the town of Glastonbury. The earliest chapel may have dated to the seventh century and religious activity persisted until the sixteenth century. A substantial cemetery is adjacent to the chapel. The site overlies interbedded mudstone (Charmouth formation) and limestone (Langport Member Blue Lias formation). Faunal remains were recovered from an evaluation by South West Heritage Trust [46]. Sampled faunal remains date from phases 3a (950 to 1150 AD) and 3 (thirteenth to sixteenth century). The assemblage is dominated by the three main domesticates with a particular focus on sheep, for both wool and meat [47].

Three sites from the Fens in eastern England were sampled from collections held at Cambridge Archaeology Unit (CAU) and included the predominantly Bronze Age site of Eye, predominantly late Iron Age site of Over, and the Late Bronze Age site at Langtoft. The Cambridgeshire Fenlands have been widely excavated and studied and are rich in archaeological wetland artefacts, notably the Bronze Age site of Flag Fen. The small village of Eye lies a few miles northeast from the city of Peterborough and 3 km from Flag Fen. Lying only a few metres above sea-level, the site was an island of higher ground within the fenland marshes, prior to the seventeenth century drainage of the fens. The site lies on Oxford Clay with some river terrace deposits. A series of excavations by CAU have uncovered a small Late Bronze Age/Early Iron Age settlement with later Romano-British activity [48]. The animal bone assemblage recorded from the Eye Quarry excavation is dominated by livestock species. The multi-period site of Over, on the banks of the River Ouse, dates from the Mesolithic to the Iron Age and has a rich and varied palaeoecology [49]. The site consists of a Fen edge, delta-complex with mid-stream islands and waterlogged barrows. Excavations have revealed varied and abundant ecofacts indicative of an intense settlement history, culminating in an Iron Age shrine. The final Fenland site of Langtoft is centred on the remains uncovered at Baston Quarry (number 2) in Lincolnshire [50]. Langtoft Fen is situated 12 km north of Peterborough, and a few km northeast from the town of Market Deeping. Baston Quarry sits on a series of alluvial gravels interspersed with palaeochannels of the River Welland. The river terrace gravels are underlain by Oxford Clay and sit a few metres in altitude above the former Fen edge to the east. Excavations by CAU have revealed a settlement, spanning later prehistory through to the Romano-British period [50].

### 2.4. Isotope methods

Plant samples were collected into paper bags and dried at 30°C overnight. The plant samples were not washed or cleaned prior to analysis as we wanted to replicate the natural biosphere conditions as much as possible. They were transferred to plastic resealable bags and crumbled, by hand, until they were ‘tea leaf’ consistency. About 1 g was transferred to a cryogenic mill where they were reduced to a powder over 1–2 min. For *δ*^34^S analysis 2 mg of powdered material was weighed into tin capsules and measured in duplicate by continuous flow-elemental analyser-isotope ratio mass spectrometry (CF-EA-IRMS) at the British Geological Survey, Keyworth UK. The instrumentation comprises a ThermoFinnigan EA IsoLink coupled to a Delta V Plus IRMS via a ConFlo IV interface. Sulfur isotope ratios (*δ*^34^S) are reported in per mil (‰) and normalized to Vienna Canyon Diablo Troilite (VCDT) using the International Atomic Energy Agency (IAEA) reference materials IAEA-S-1 (silver sulfide, *δ*^34^S_VCDT_ = –0.30‰), IAEA-S-2 (silver sulfide, *δ*^34^S_VCDT_ = + 22.66‰), IAEA-S-3 (silver sulfide, *δ*^34^S_VCDT_ = –32.49‰). Two in-house standards (BROC2, *δ*^34^S_VCDT_ = + 11.55‰ ± 0.29‰, *n* = 8) and elemental microanalysis spirulina standard (B2162, *δ*^34^S_VCDT_ = + 13.53 ± 0.17‰, *n* = 4) that are independently calibrated to the IAEA reference materials IAEA-S-1, IAEA-S-2 and IAEA-S-3, were used as a secondary check standards. All samples were analysed in duplicate and gave an average 1 s reproducibility of ±0.3‰. Weight % sulfur was calculated using an in-house broccoli standard (BROC2, S% = 0.84) calibrated using SOIL A (LECO – part number 502-309). Results are reported as per mil (‰) relative to the internationally accepted standard VCDT.

Modern faunal samples were defatted using a 1 : 2 methanol and chloroform solvent and placed in an ultrasonic bath for 30 min. This process was repeated at least three times, replacing the solvent until all lipids were removed. Samples were then rinsed three times in methanol and allowed to dry at room temperature. Approximately 0.5 g of modern and archaeological bone samples were demineralized in 0.5 M HCl at 5°C. Following demineralization, samples were gelatinized in pH 3 HCl at 70°C in a hot block before freeze drying. Stable carbon (*δ*^13^C), nitrogen (*δ*^15^N), and sulfur (*δ*^34^S) isotopic compositions were determined on a Delta V Advantage CF-IRMS coupled via a ConfloIV to an IsoLink EA (Thermo Scientific, Bremen) at SUERC, East Kilbride as described in Sayle *et al.* [25]. The IAEA reference materials USGS40 (l-glutamic acid, *δ*^13^C_VPDB_ = –26.39 ± 0.04‰, *δ*^15^N_AIR_ = –4.52 ± 0.06‰) and USGS41a (l-glutamic acid, *δ*^13^C_VPDB_ = + 36.55 ± 0.08‰, *δ*^15^N_AIR_ = + 47.55 ± 0.15‰) were used to normalize *δ*^13^C and *δ*^15^N values. Two in-house standards (GS2, *δ*^34^S_VCDT_ = –10.28 ± 0.18‰ and GAS2, *δ*^34^S_VCDT_ = + 18.56 ± 0.10‰) that are calibrated to the IAEA reference materials IAEA-S-2 (silver sulfide, *δ*^34^S_VCDT_ = + 22.62 ± 0.08‰) and IAEA-S-3 (silver sulfide, *δ*^34^S_VCDT_ = –32.49 ± 0.08‰) were used to normalize *δ*^34^S values. Results are reported as per mil (‰) relative to the internationally accepted standards Vienna Pee Dee Belemnite (VPDB), Air (AIR) and VCDT. See the electronic supplementary material, table S3 for further details of normalization methods. The collagen atomic carbon/nitrogen ratios fall into the expected range for well-preserved collagen (table 2; 2.9–3.6; [51]) with two exceptions (OVE11-1089-SG and MU24). Criteria for assessing sulfur preservation in collagen are less well established but [52] suggest that atomic carbon/sulfur ratios between 300–900 and atomic nitrogen/sulfur ratios between 100 and 300 were indicative of well-preserved collagen.

**Table 2.** Carbon (C), nitrogen (N) and sulfur (S) isotope composition of modern and archaeological fauna collected from wetland regions in southern England.

site	sample ID	*δ*¹⁵N_AIR_ ‰	*δ*¹³C_VPDB_ ‰	*δ*³⁴S_VCDT_ ‰	%N	%C	%S	C/N at	N/S at	C/S at
Beckery	BK27	11.4	−22.1	−10.6	6.8	20.2	0.18	3.48	87	300
Beckery	BK25	9.2	−22.2	0.2	14.0	40.5	0.22	3.38	146	492
Beckery	BK26	7.1	−22.1	8.7	13.4	38.5	0.19	3.36	164	549
Beckery	BK28	7.3	−21.7	−1.2	11.9	34.3	0.19	3.37	143	482
Beckery	BK29	4.1	−22.1	11.8	9.5	26.4	0.14	3.25	155	503
Beckery	BK30	6.4	−21.3	−5.8	9.7	26.6	0.14	3.21	159	507
Beckery	BK31	10.3	−22.1	−0.6	15.1	41.9	0.20	3.25	173	559
Beckery	BK32	3.7	−21.8	11.7	13.0	36.4	0.17	3.28	173	566
Beckery	BK33	8.8	−22.5	4.4	13.3	38.4	0.19	3.38	160	540
Beckery	BK34	8.1	−21.2	−6.9	15.5	43.7	0.21	3.30	169	556
Beckery	BK35	8.4	−21.3	−6.4	13.1	37.6	0.17	3.36	176	591
Beckery	BK36	11.7	−22.6	−0.4	13.1	37.2	0.20	3.32	150	497
Burtle Priory	BP01	6.9	−22.8	7.9	7.4	20.6	0.12	3.26	141	458
Burtle Priory	BP02	10.8	−−21.8	7.6	12.8	36.5	0.17	3.34	172	573
Burtle Priory	BP03	8.7	−21.9	8.1	11.0	30.1	0.14	3.20	180	574
Burtle Priory	BP04	9.8	−22.1	4.0	13.7	38.9	0.20	3.32	156	519
Burtle Priory	BP05	9.1	−21.6	4.9	15.0	42.2	0.20	3.29	172	563
Burtle Priory	BP06	6.7	−23.9	2.4	3.4	10.6	0.07	3.65	111	404
Burtle Priory	BP07	7.2	−23.4	8.1	2.7	8.3	0.07	3.60	88	317
Burtle Priory	BP08	6.9	−21.6	5.8	11.6	31.6	0.17	3.19	156	496
Burtle Priory	BP09	9.6	−22.3	8.1	7.8	21.0	0.10	3.15	179	561
Burtle Priory	BP10	11.7	−23.0	3.9	14.1	39.8	0.19	3.30	170	559
Burtle Priory	BP11	5.3	−22.4	13.8	9.0	25.5	0.14	3.32	147	486
Burtle Priory	BP12	10.3	−23.4	7.0	13.6	39.5	0.21	3.40	148	502
Eye	EYE06-264-C	5.9	−22.1	1.4	15.3	44.0	0.24	3.36	146	490
Eye	EYE06-264-SG	4.8	−22.6	−9.5	15.8	45.2	0.21	3.35	173	575
Eye	EYE06-457-C	7.0	−22.6	0.4	15.8	45.2	0.21	3.35	172	575
Eye	EYE06-636-SG	7.8	−21.6	−13.4	13.4	39.0	0.25	3.41	123	417
Eye	EYE06-654-C	6.4	−22.3	−11.0	14.6	41.4	0.20	3.32	167	553
Eye	EYE06-663-C	6.2	−22.4	−5.2	13.7	38.3	0.19	3.27	165	538
Eye	EYE06-663-SG	5.5	−22.2	−3.4	15.1	43.2	0.19	3.35	182	607
Eye	EYE06-972-C	6.8	−21.8	−8.3	9.6	26.4	0.15	3.22	147	470
Eye	EYE06-994-SG	7.0	−21.7	−9.8	15.3	43.7	0.27	3.34	130	432
Eye	EYE06-995-SG	6.2	−22.2	−7.0	15.6	45.1	0.27	3.38	132	446
Langtoft	LAN98-167-SG	8.1	−22.5	−4.0	11.3	31.3	0.16	3.24	162	522
Langtoft	LAN98-172-C	4.0	−22.7	6.5	6.7	18.1	0.10	3.16	153	483
Langtoft	LAN98-173-C	5.6	−22.6	4.9	5.3	14.7	0.11	3.25	110	357
Langtoft	LAN98-187-SG	6.0	−23.9	−5.8	8.0	21.6	0.11	3.16	167	524
Langtoft	LAN98-200-C	6.0	−22.1	−15.2	14.8	41.5	0.30	3.28	113	369
Langtoft	LAN98-235-C	6.2	−23.5	1.7	15.1	42.4	0.22	3.29	157	515
Langtoft	LAN98-255-SG	5.6	−21.6	1.2	15.6	44.5	0.20	3.34	179	594
Muchelney	MU13	8.2	−22.3	2.4	13.7	38.7	0.18	3.31	174	574
Muchelney	MU14	8.6	−22.2	−8.5	14.6	40.9	0.20	3.28	167	546
Muchelney	MU15	8.5	−21.6	−1.3	13.1	36.1	0.17	3.22	176	567
Muchelney	MU16	6.8	−22.1	5.0	13.4	37.5	0.17	3.27	181	588
Muchelney	MU17	7.7	−21.3	−7.0	14.7	41.3	0.21	3.29	160	525
Muchelney	MU18	9.9	−22.4	−8.7	13.3	38.2	0.19	3.36	160	537
Muchelney	MU19	6.8	−22.3	8.3	10.9	30.1	0.15	3.23	166	536
Muchelney	MU20	8.9	−22.0	−5.7	10.3	28.3	0.15	3.21	157	504
Muchelney	MU21	7.9	−22.3	−8.3	9.4	26.3	0.14	3.27	154	502
Muchelney	MU22	7.9	−22.4	−4.7	10.1	28.3	0.15	3.28	154	504
Muchelney	MU23	8.2	−22.1	−12.7	11.4	31.4	0.16	3.22	163	524
Muchelney	MU24	7.5	−22.7	−9.9	3.3	8.7	0.09	3.08	84	258
Over	OVE07-3221-C	7.9	−21.5	−8.0	16.5	45.5	0.25	3.23	151	486
Over	OVE07-3226-C	7.4	−21.7	−10.8	15.9	44.3	0.26	3.26	140	455
Over	OVE07-3226-SG	6.8	−21.7	−13.8	16.2	45.4	0.24	3.28	155	505
Over	OVE07-322-SG	5.9	−21.4	−12.4	15.8	45.7	0.25	3.38	145	488
Over	OVE07-3235-SG	5.5	−21.0	−15.6	15.7	42.8	0.25	3.19	143	457
Over	OVE07-3242-C	4.9	−21.2	−3.5	16.1	46.4	0.26	3.37	142	476
Over	OVE07-3335-C	5.8	−21.5	−13.0	15.6	42.9	0.22	3.22	162	521
Over	OVE07-3335-SG	6.4	−22.1	−6.7	16.3	45.7	0.25	3.28	149	488
Over	OVE08-1997-SG	8.4	−22.0	−19.7	15.7	43.7	0.27	3.26	133	432
Over	OVE08-398-C	5.2	−21.5	−6.9	10.9	30.3	0.16	3.25	156	506
Over	OVE11-1089-SG	8.0	−22.4	−16.5	13.5	38.6	0.39	3.35	79	264
Over	OVE11-1092-C	9.3	−22.1	−16.1	15.6	44.3	0.27	3.32	132	438
modern										
Peterborough	BAP-08-A-1	7.3	−24.8	−9.5	14.7	41.8	0.25	3.33	135	446
Peterborough	BAP-08-B-1	7.4	−24.4	−9.5	16.2	45.5	0.28	3.29	133	434
Peterborough	BAP-09a	8.6	−23.4	−12.5	16.2	45.1	0.23	3.26	161	524
Peterborough	BAP-09b	9.3	−24.0	−13.3	15.7	45.9	0.24	3.42	150	511
Somerset	SL01	6.6	−24.8	3.1	15.7	43.5	0.19	3.24	189	611
Somerset	SL02	8.6	−24.8	2.1	16.2	44.6	0.19	3.22	195	627
Somerset	SL03	6.5	−24.6	2.8	16.1	45.1	0.19	3.28	194	634
Somerset	SL04	7.5	−25.4	3.0	15.3	42.8	0.20	3.27	175	571
Somerset	SL-14	7.4	−25.2	4.9	15.8	45.1	0.28	3.34	129	430
Somerset	SL-25-A-1	7.7	−22.5	4.5	16.2	45.5	0.28	3.29	132	434
Somerset	SL-25-B-1	7.0	−23.1	5.0	16.3	44.8	0.24	3.22	156	498

## 3. Results

### 3.1. Modern plants

**Figure 2.**
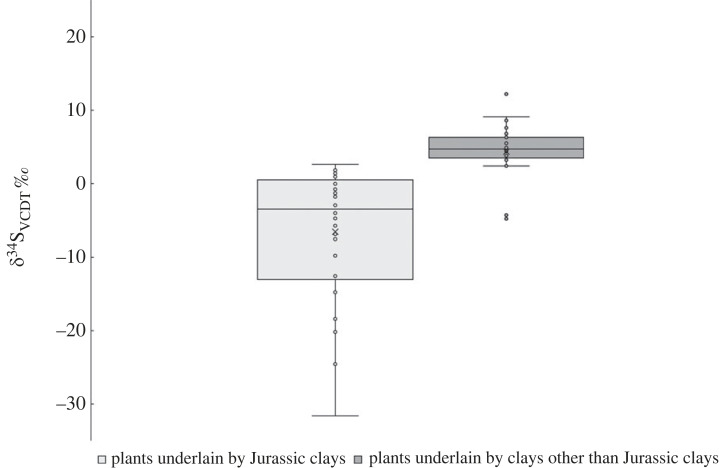
A comparison of *δ*^34^S values from plants grown on Jurassic clays and plants grown on other substrates (other clay lithologies: data from Chenery *et al.* [53], Evans *et al.* [54]). Box and whisker plot constructed exclusive of median.

The *δ*^34^S results from plants collected in a number of wetland regions of southern England are given in table 1 and figure 1. The data from this study support the founding observation that plants growing on Jurassic clay give significantly lower sulfur isotope values than on other substrates. Figure 2 compares sulfur isotope values from plants collected in this study growing on Jurassic clay bedrock with plants from previously collected samples [53,54] from non-Jurassic clay bedrock. The Jurassic clay hosted plants record an average sulfur isotope value of –5.8 ± 8.8‰ (1 s.d., *n* = 58), whereas the sample derived from a range of other clay lithologies (Ordovician, Silurian Devonian, Triassic and Cretaceous) averages +4.4 ± 3.7‰ (1 s.d., *n*= 29) (data from [53], V1 data spreadsheet).

### 3.2. Relationship between altitude and sulfur isotope compositions

**Figure 3.**
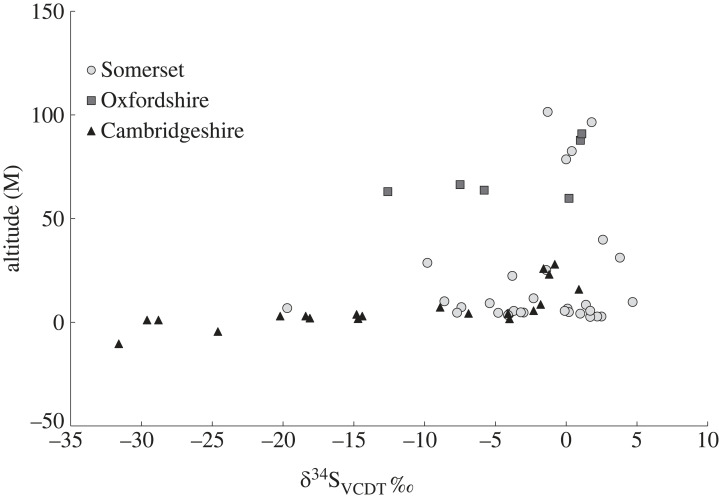
A comparison of plant sulfur isotope values with altitude.

Figure 3 shows the sulfur isotope composition of the plant samples plotted against the altitude at which they were collected. All sites show a broadly similar pattern of the highest sulfur isotope values being found at the highest altitudes, a rapid fall of sulfur isotope values with altitude and then an extension of sulfur isotope values down to low and very low values at the lowest altitudes at each site. The majority of the data producing negative sulfur isotope values come from sites below 20 m altitude; the Oxfordshire data is different with its extension to low sulfur values occurring at *ca* 60 m altitude, however, this is likely to be a result of differences in topography and altitudinal range between the locations. It is to be noted that for the areas in Cambridgeshire, sampling was undertaken in areas where waterlogged soil conditions still persist, whereas the Somerset Levels and the areas south of Oxfordshire are currently moderately well drained. This may largely account for the lower sulfur isotope values coming predominantly from Cambridgeshire. The issues of the changed environmental conditions between the modern and the historical periods cannot be resolved using the plant data, but animal bone collagen (ancient and modern) was analysed to assess (i) whether the transmission of these low plant sulfur isotope values is seen in the fauna grazing such areas, and (ii) whether we could see a difference in ancient and modern animal sulfur isotope values.

### 3.3. Fauna

**Figure 4.**
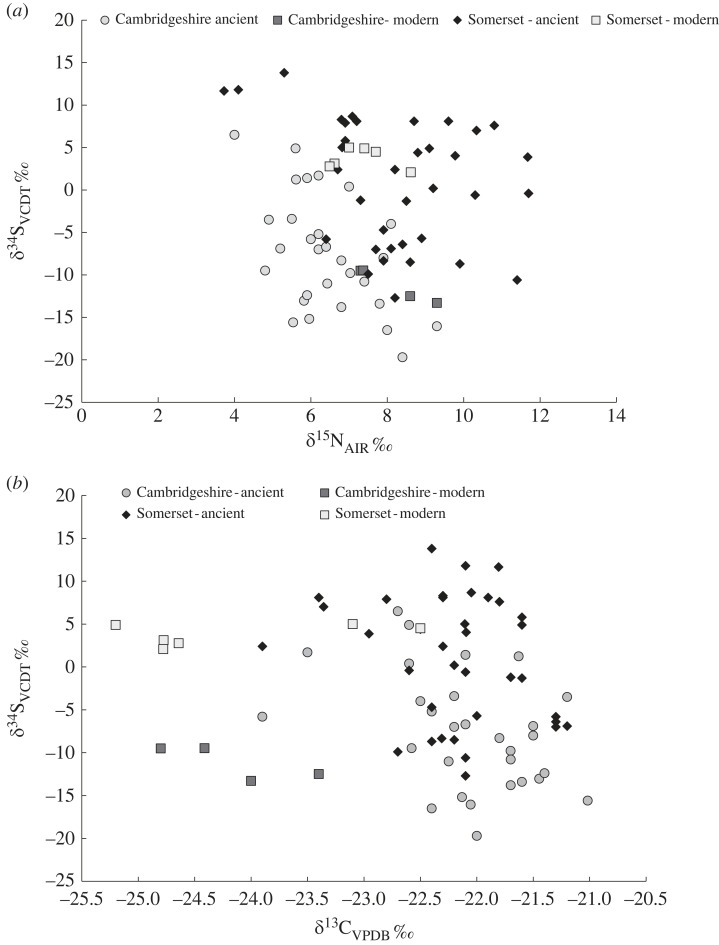
Sulfur isotope composition of bone collagen plotted against (*a*) nitrogen isotope composition and (*b*) carbon isotope composition from the two main wetland regions: Somerset and Cambridgeshire.

Sixty-four samples of collagen from archaeological sheep and cattle bone (29 from Cambridgeshire and 36 from Somerset), and 11 samples of collagen from modern sheep and cattle (four from Cambridgeshire and seven from Somerset) were analysed for sulfur, carbon and nitrogen isotope composition (table 2). The data are shown on two diagrams plotting figure 4*a δ*^34^S_VCDT_ versus *δ*^15^N_AIR_ and figure 4*b*, *δ*^34^S_VCDT_ versus *δ*^13^C_VPDB_. Several features can be seen in these plots. Sixty per cent of the archaeological faunal samples produced negative sulfur isotope values with the data from Cambridgeshire (mean –7.6 ± 6.8‰, *n* = 29) substantially lower that Somerset (mean +1.0 ± 7.4‰, *n* = 36). Cambridgeshire archaeological fauna also show a more restricted range and lower average *δ*^15^N_AIR_ values (+6.4 ± 1.2‰, *n* = 29) compared with Somerset (+8.2 ± 1.9‰, *n* = 36). This pattern is reversed in the modern data where the modern Cambridgeshire animals have a mean *δ*^15^N_AIR_ of +8.4 ± 1.0‰ (*n* = 4) and Somerset of +7.3 ± 0.7‰ (*n* = 7) but sample size is small and this may reflect differences in husbandry and foddering. For carbon isotopes, archaeological fauna are indistinguishable with *δ*^13^C_VPDB_ values for Cambridgeshire (–22.1 ± 0.6‰, *n* = 29) and Somerset (–22.2 ± 0.6‰, *n* = 36) virtually identical. This is replicated in the modern data where the Cambridgeshire animals have a mean *δ*^13^C_VPDB_ of –24.1 ± 0.6‰ (*n* = 4) and Somerset of –24.3 ± 1.1‰ (*n* = 7), with the approximately 2‰ lower *δ*^13^C_VPDB_ values in the modern animals from both locations probably reflecting the effect of fossil fuel burning on atmospheric *δ*^13^C_VPDB_ values [55].

Of significance is that the modern fauna collected from Cambridgeshire and Somerset have sulfur isotope differences that mirror the archaeological fauna (Cambridgeshire mean –11.8 ± 2.0‰, *n* = 4), being significantly lower that the animals raised in Somerset (mean +3.6 ± 1.2‰, *n* = 7). The observation that the modern animal sulfur isotope data plot within the field of the archaeological samples supports the argument that these negative sulfur isotope collagen values are a primary feature of the environment and not overprinted by modern pollution. There is a clear geographical divide in both ancient and modern animal collagen *δ*^34^S_VCDT_ groupings but this geographical divide is not evident in the carbon and nitrogen data. The majority of modern animal samples were taken from organic farms; this means the meat is grown according to organic food principles- this does not necessarily equate to a natural environment.

Samples of both archaeological sheep and cattle were taken but there is no overall statistical difference between the cattle and sheep sulfur isotope data when both locations are compared (*Bos* –2.8 ± 7.9‰, *n* = 33, and *Ovis* –2.8 ± 8.8‰, *n* = 32) and this is the case for carbon (*Bos* –22.1 ± 0.7‰, *n* = 33, and *Ovis* –22.2 ± 0.6‰, *n* = 32) and nitrogen (*Bos* +7.1 ± 1.7‰, *n* = 33, and *Ovis* +7.7 ± 2.0‰, *n* = 32) isotope values. When coupled with the observation that there is a correlation between altitude and plant sulfur values, this would indicate that the animals were not segregated on pastures relative to altitude. In other words, there is no evidence that sheep graze on higher pasture that cattle.

## 4. Discussion

The aim of this study was to assess whether there is a relationship between biosphere sulfur isotope characteristics and Jurassic clay lithology, the primary lithology that supports wetland regions in Britain. Erosion of these clays will, thus, supply reduced sulfur into the soil profile. Our hypothesis is that the Jurassic clays provide two drivers for such a relationship: (i) that the Jurassic clays contain high levels of reduced sulfides derived from the anaerobic conditions in which the clay formed. Jurassic clays have a high pyrite content, which frequently preserves fossils [56], caused by the largely anaerobic conditions during deposition [57]. Pyrite sulfur isotope compositions have been shown to produce extremely low *δ*^34^S values, for example within the Oxford Clay: −12 to −46‰ [58] and within the early Jurassic Blue Lias formation in Dorset, southern England (−36 to −46‰, [59]); and (ii) the impervious nature of the clays means that their overlying soils are also commonly waterlogged, leading to anaerobic conditions and supporting widespread wetland development, so both the inherited sulfur and any formed in the soil surface environment will drive down sulfur isotope values in the biosphere. Both the primary sulfide composition of the clays and the anaerobic conditions generated in the supported wetlands will lead to decreasing sulfur isotope values that are transmitted into the biosphere. Which of these processes is dominant is difficult to ascertain, however as sulfides are toxic to most plants, the incorporation of reduced sulfur into the biosphere in non-tidal wetland environments is likely to be dominated by the substrate baseline, with sulfide uptake driving this process further where sulfide-adapted plants are present and thus able to transfer to the biosphere. The significance of this relationship to the substrate, if shown, is that wetland low sulfur isotope values in biosphere transmitted ecosystems can be used as a proxy for wetland habitation in the past. Wider understanding of how sulfides (or sulfates following intermediate reoxidation) are incorporated into the biosphere and into food webs is needed to disentangle the mechanisms leading to the incorporation of both low and variable biosphere *δ*^34^S values.

We sampled plants from two widescale areas of wetland on the Jurassic clays: the eastern end of the outcrop which incorporated areas of Cambridgeshire and the western end that includes the Somerset Levels. We also had a small plant sample set from between the two areas, in Oxfordshire. We also took samples from archaeological animal samples from the two sites and modern organically raised animals in order to examine the transmission of isotope signals through the food chain and compare modern and ancient animal values. A comparison of plant data from the Jurassic clays with other English clay outcrops confirms that the Jurassic clays support lower sulfur isotope values than other English clay outcrops (figure 2). There are differences between the Somerset and the Cambridgeshire area with the latter supporting the lowest biosphere sulfur isotope values. It could be conjectured that this difference in modern plants is owing to the Somerset Levels now being drained, but the difference is seen in the archaeological animal data as well as the modern data and so this explanation seems unlikely. An alternative possibility is that the Somerset Levels are close to the coast and were periodically inundated with marine water which will introduce significantly elevated ^34^S into the area [60]. In some cases, marine water has reached as far inland as Glastonbury, owing to extreme marine incursions such as the 1607 tsunami event [61,62]. Another significant difference between the two areas is that the Somerset Levels have areas of land that remain elevated above the wetlands. The effect of this is seen in figure 3 where the plant samples from higher altitudes have higher sulfur isotope values and it is possible that the higher land was used for both crops and grazing. Sulfur isotope values from archaeological sheep and cattle, show no systematic difference in composition, indicating there was no stratification of the grazing between the species.

Sulfur isotopes can therefore potentially be used as a diagnostic proxy in archaeological studies for characterizing fauna (and humans) that have been raised on or used wetland environments. This observation unleashes the potential for exploring past rural networks that involve these fertile areas.

**Figure 5.**
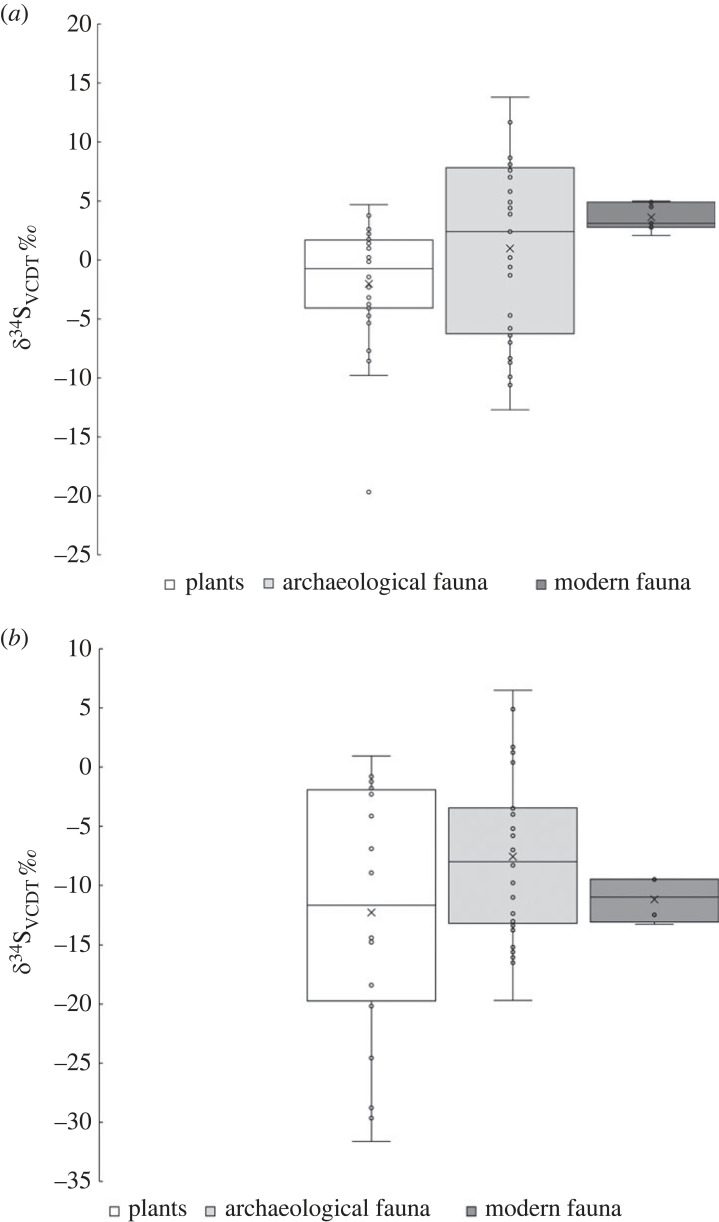
A comparison of archaeological faunal sulfur values with modern plants and fauna from (*a*) Somerset and (*b*) Cambridgeshire. Box and whisker plots constructed exclusive of median.

The negative sulfur values previously reported by [15,37,62] all come from areas underlain by Jurassic clays. This both supports the assertion that this lithology is a major source of negative sulfur and could provide evidence of locally dwelling populations where the local biosphere conditions are derived from Jurassic clay lithologies. A comparison of sulfur isotope ranges for modern plants and animals with archaeological samples, from the two main locations in the study of Somerset (figure 5*a*) and Cambridgeshire (figure 5*b*), shows that in both cases the modern data is consistent with the archaeological data that represent the environmental composition before modern pollution. We would not expect to see a relationship between altitude and sulfur isotope values if the data were because of blanket modern pollution deposition (figure 3). The wider implications of this study are that modern pollution has been reduced to a point where the measurement of sulfur in modern plants can be used as a proxy for past environment. However, there will be places where modern pollutants still contribute to the biosphere so care should be taken where selecting sample sites and known areas of heavy pollution should be avoided. The sites examined here are rural locations in the southwest and east of England and may not fully represent the wider pollution picture in the UK. Zhao and colleagues [28] have demonstrated that SO_2_ emissions have declined rapidly, accompanied by an increase in *δ*^34^S values (following a change in UK legislation with The Clean Air Act of 1956). This has paved the way for detailed *δ*^34^S biosphere maps to be developed [38] that can be used for exploring origins and mobility in humans and animals. It also means that low and negative values which would have previously been considered to result from pollution, now have the potential to be used as a diagnostic signature for identifying origins in waterlogged terrains. However, the impact of pollution is not clear cut and a better understanding of how ecosystems respond to fluctuations in drivers such as changes in air pollution would improve the validity of sulfur isotopes as a proxy for past hydrological conditions.

Using the data and conclusions of this study, a sulfur isotope map of Britain has been produced [38]. This map presents variations in modern plant *δ*^34^S across Britain and may be useful, not only in showing the broad trends of *δ*^34^S plant values across Great Britain, including the low *δ*^34^S zone over the Jurassic clays, but for use as a guide for human and animal mobility studies. However, when extending the application of these plant data to archaeological studies of humans and animals, other factors will need to be taken into consideration. Unlike strontium isotopes (^87^Sr/^86^Sr), which are released into the biosphere by erosion and are unaffected by fractionation during this process, sulfur isotopes will undergo fractionation in response to local surface conditions. Therefore, the link between geology and biosphere is far looser than for strontium isotope systems. A further factor in the application of sulfur as a geographical indication of origin is that, whereas strontium consumed by humans is most strongly controlled by the plants/cereals they consume, the main sources of sulfur to humans are more variable and can be dominated by animal protein sources so the use of plant mapping to source humans may, again, be less direct. Finally, although there is evidence to support declining levels of SO_2_ atmospheric pollution in the UK, this study focussed on two rural regions in England and the apparent lack of atmospheric pollutants evident here within the local biosphere, may not be mirrored across other, more industrialised, areas.

## 5. Conclusion

This study has shown that there is a spatial correlation between the Jurassic clay outcrop in southern Britain and the occurrence of relatively low or negative *δ*^34^S values in modern plants collected within this geological domain. Sixty per cent of the samples gave values below zero. The modern plant datasets give more negative values for the eastern regions of Cambridgeshire relative to Oxfordshire and Somerset. Collagen from archaeological samples of bone collected from Somerset and Cambridgeshire also show a substantial percentage of negative sulfur isotope values (60.3%), again, with more negative values from the Cambridgeshire sites as does modern collagen. The plants show a correlation between sulfur isotope composition and altitude which supports low lying wetlands supplying the most negative values into the biosphere, but there is no evidence of such altitude stratification in the animals so it must be assumed that cattle and sheep grazed similar pastures. A *δ*^34^S value of zero or below is a convenient point to ascribe to the dataset presented here, however we would like to stress that this is not a marker for describing wetland environments and may vary between sites and paleoenvironmental conditions. Further work is needed to resolve regional differences in the altitude below which low values occur and also to understand sulfur isotope variability in higher altitude locations on the Jurassic clay.

These results support the interpretation that relatively low or negative sulfur isotope values are indicative of flora and fauna growing and grazing on wetland regions underlain by Jurassic clays. We suggest that this is owing both to the origin of the substrate and its impermeability, producing redox conditions and sulfide-rich anaerobic wetlands. The disseminated sulfides in bedrock, soils and groundwater of Jurassic clay lithologies, as a result, produce significantly lower biosphere sulfur isotope values through the activity of phototropic sulfur bacteria and a significant kinetic isotope effect. The sulfides produced are depleted in ^34^S relative to the substrate and total soil sulfur becomes progressively more ^34^S depleted as sulfates are removed [5]. This study provides a provenancing approach for characterizing collagen with low sulfur isotope values as diagnostic of wetland conditions, and in Britain most found on Jurassic clays terrain. As a result, archaeological humans and animals from wetlands (or that acquired their food from wetlands) may be identified using primary analytical methods. This provides an additional tool for archaeologists to explore animal management and human and animal mobility in the past.

## Data Availability

The datasets supporting this article have been uploaded as part of the electronic supplementary material [63].

## References

[1] Nehlich O. 2015 The application of sulphur isotope analyses in archaeological research: a review. Earth Sci. Rev. **142**, 1-17. (10.1016/j.earscirev.2014.12.002)

[2] Krajcarz MT, Krajcarz M, Drucker DG, Bocherens H. 2019 Prey-to-fox isotopic enrichment of 34S in bone collagen: implications for paleoecological studies. Rapid Commun. Mass Spectrom. **33**, 1311-1317. (10.1002/rcm.8471) 31017708

[3] McArdle N, Liss P, Dennis P. 1998 An isotopic study of atmospheric sulphur at three sites in Wales and at Mace Head, Eire. J. Geophys. Res. **103**, 31 079-31 094. (10.1029/98JD01664)

[4] Trust BA, Fry A. 1992 Stable sulphur isotopes in plants: a review. Plant Cell Environ. **15**, 1105-1110. (10.1111/j.1365-3040.1992.tb01661.x)

[5] Krouse HR. 1989 Sulfur isotope studies of the pedosphere and biosphere. In Stable isotopes in ecological research (eds PW Rundel, JR Ehleringer, KA Nagy), pp. 424-444. New York, NY: Springer.

[6] Fry B, Scalan RS, Winters JK, Parker PL. 1982 Sulfur uptake by salt grasses, mangroves, and seagrasses in anaerobic sediments. Geochim. Cosmochim. Acta **46**, 1121-1124. (10.1016/0016-7037(82)90063-1)

[7] Peterson BJ, Fry B. 1987 Stable isotopes in ecosystem studies. Annu. Rev. Ecol. Syst. **18**, 293-320. (10.1146/annurev.es.18.110187.001453)

[8] Lamers LPM, Govers LL, Janssen ICJM, Geurts JJM, Van Der Welle MEW, Van Katwijk MM. 2013 Sulfide as a soil phytotoxin - a review. Front. Plant Sci. **4**, 268. (10.3389/fpls.2013.00268) 23885259 PMC3717504

[9] Carlson PR, Forrest J. 1982 Uptake of dissolved sulfide by *Spartina alterniflora*: evidence from natural sulfur isotope abundance ratios. Science **216**, 633-635. 17783311 10.1126/science.216.4546.633

[10] Cornwell JC, Neill C, Stevenson JC. 1995 Biogeochemical origin of *δ*^34^S isotopic signatures in a prairie marsh. Can. J. Fish. Aquat. Sci. **52**, 1816-1820.

[11] Guiry EJ, Orchard TJ, Needs-Howarth S, Szpak P. 2022 Freshwater wetland–driven variation in sulfur isotope compositions: implications for human paleodiet and ecological research. Front. Ecol. Evol. **10**, 953042. (10.3389/fevo.2022.953042)

[12] Hebert CE, Wassenaar LI. 2005 Feather stable isotopes in western North American waterfowl: spatial patterns, underlying factors and management applications. Wildlife Soc. Bull. **33**, 92-102. (https://www.jstor.org/stable/3784844)

[13] Guiry E, Noël S, Fowler J. 2021 Archaeological herbivore *δ*^13^C and *δ*^34^S provide a marker for saltmarsh use and new insights into the process of 15N-enrichment in coastal plants. J. Archaeol. Sci. **125**, 105295. (10.1016/j.jas.2020.105295)

[14] Stevens RE, Reade H, Read DS, Bottrell SH, Frémondeau D, Wexler S. 2022 Iso-wetlands: unlocking wetland ecologies and agriculture in prehistory through sulfur isotopes. Archaeol. Int. **25**, 168-176. (10.14324/111.444.ai.2022.11)

[15] Nehlich O, Borić D, Stefanović S, Richards MP. 2010 Sulphur isotope evidence for freshwater fish consumption: a case study from the Danube Gorges, SE Europe. J. Archaeol. Sci.: Rep. **37**, 1131-1139. (10.1016/j.jas.2009.12.013)

[16] Privat KL, O'Connell TC, Hedges REM. 2007 The distinction between freshwater-and terrestrial-based diets: methodological concerns and archaeological applications of sulphur stable isotope analysis. J. Archaeol. Sci. **34**, 1197-1204. (10.1016/j.jas.2006.10.008)

[17] Bocherens H, Drucker DG, Taubald H. 2011 Preservation of bone collagen sulphur isotopic compositions in an early Holocene river-bank archaeological site. Palaeogeogr. Palaeoclimatol. Palaeoecol. **310**, 32-38. (10.1016/j.palaeo.2011.05.016)

[18] Craig OE, Ross R, Andersen SH, Milner N, Bailey GN. 2006 Focus: sulphur isotope variation in archaeological marine fauna from northern Europe. J. Arch. Sci. **33**, 1642-1646. (10.1016/j.jas.2006.05.006)

[19] Madgwick R, Sykes N, Miller H, Symmons R, Morris J, Lamb A. 2013 Fallow deer (*Dama dama dama*) management in Roman south-east Britain. Archaeol. Anthropol. Sci. **5**, 111-122. (10.1007/s12520-013-0120-0)

[20] Lamb AL, Melikian M, Ives R, Evans J. 2012 Multi-isotope analysis of the population of the lost medieval village of Auldhame, East Lothian, Scotland. J. Anal. At. Spectrom. **27**, 765-777. (10.1039/c2ja10363j)

[21] Madgwick R, Grimes V, Lamb AL, Nederbragt AJ, Evans JA, McCormick F. 2019 Feasting and mobility in Iron Age Ireland: multi-isotope analysis reveals the vast catchment of Navan Fort, Ulster. Sci. Rep. **9**, 19792. (10.1038/s41598-019-55671-0) 31874966 PMC6930251

[22] Madgwick R, Lamb AL, Sloane H, Nederbragt AJ, Albarella U, Pearson MP, Evans JA. 2019 Multi-isotope analysis reveals that feasts in the Stonehenge environs and across Wessex drew people and animals from throughout Britain. Sci. Adv. **5**, eaau6078. (10.1126/sciadv.aau6078) 30891495 PMC6415963

[23] Madgwick R, Lamb A, Sloane H, Nederbragt A, Albarella U, Parker Pearson M, Evans J. 2021 A veritable confusion: use and abuse of isotope analysis in archaeology. Archaeological J. **178**, 361-385. (10.1080/00665983.2021.1911099)

[24] Scorrer J, Faillace KE, Hildred A, Nederbragt AJ, Andersen MB, Millet M-A, Lamb AL, Madgwick R. 2021 Diversity aboard a Tudor warship: investigating the origins of the Mary Rose crew using multi-isotope analysis R. Soc. open sci. **8**, 202106202106. (10.1098/rsos.202106) PMC8097207 34035946

[25] Sayle KL, Brodie CR, Cook GT, Hamilton WD. 2019 Sequential measurement of *δ*15 N, *δ*13 C and *δ*34 S values in archaeological bone collagen at the Scottish Universities Environmental Research Centre (SUERC): A new analytical frontier. Rapid Commun. Mass Spectrom. **33**, 1258-1266. (10.1002/rcm.8462) 30993809

[26] Nriagu J, Rees CE, Mekhtieva VL, Lein AY, Fritz P, Drimmie RJ, Pankina RG, Robinson RW, Krouse HR. 1991 Hydrosphere. In Stable isotopes: natural and anthropogenic sulphur in the environment (eds HR Krouse, VA Grinenko), pp. 177-265. Chichester, UK: John Wiley & Sons.

[27] Richards MP, Fuller BT, Hedges REM. 2001 Sulphur isotopic variation in ancient bone collagen from Europe: implications for human palaeodiet, residence mobility and modern pollutant studies. Earth Planet. Sci. Lett. **191**, 185-190. (10.1016/S0012-821X(01)00427-7)

[28] Zhao F, Spiro B, Poulton PR, McGrath SP. 1998 Use of sulfur isotope ratios to determine anthropogenic sulfur signals in a grassland ecosystem. Environ. Sci. Technol. **32**, 2288-2291. (10.1021/ES980157F)

[29] Zhao F, Knights JS, Hu ZY, McGrath SP. 2003 Stable sulfur isotope ratio indicates long-term changes in sulfur deposition in the Broadbalk experiment since 1845. J. Environ. Qual. **32**, 33-39. (10.2134/jeq2003.3300) 12549539

[30] Krouse HR, Mayer B. 2000 Sulphur and oxygen isotopes in sulphate. In Environmental tracers in subsurface hydrology (eds PG Cook, AL Herczeg), pp. 195-231. Boston, MA: Springer.

[31] Strauch G, Schreck P, Nardin G, Gehre M. 2001 Origin and distribution of sulphate in surface waters of the Mansfeld mining district (Central Germany)—a sulphur isotope study. Isot. Environ. Health Stud. **37**, 101-112. (10.1080/10256010108033287) 11761400

[32] Nehlich O, Fuller BT, Jay M, Mora A, Nicholson RA, Smith CI, Richards MP. 2011 Application of sulphur isotope ratios to examine weaning patterns and freshwater fish consumption in Roman Oxfordshire, UK. Geochim. Cosmochim. Acta **75**, 4963-4977. (10.1016/j.gca.2011.06.009)

[33] DEFRA. 2018 Air Pollution in the UK 2017, Department for Environment, Food and Rural Affairs: ‘Air Pollution in the UK’ series of reports (online). See https://uk-air.defra.gov.uk/library/annualreport/index.

[34] Bottrell S, Coulson J, Spence M, Roworth P, Novak M, Forbes L. 2004 Impacts of pollutant loading, climate variability and site management on the surface water quality of a lowland raised bog, Thorne Moors, E. England, UK. Appl. Geochem. **19**, 413-422.

[35] Madgwick R, Esposito C, Lamb AL. 2023 Farming and feasting during the Bronze Age-Iron Age transition in Britain (ca. 900-500 BCE): multi-isotope evidence for societal change. Front. Environ. Archaeol. **2**, 1221581. (10.3389/fearc.2023.1221581)

[36] Towers J, Montgomery J, Evans J, Jay M, Parker Pearson M. 2010 An investigation of the origins of cattle and aurochs deposited in the Early Bronze Age barrows at Gayhurst and Irthlingborough. J. Archaeol. Sci. **37**, 508-515. (10.1016/j.jas.2009.10.012)

[37] Jay M, Nehlich O, Richards M. 2019 Sulphur isotope analysis. In The Beaker People: isotopes, mobility and diet in prehistoric Britain (eds M Parker-Pearson, JA Sheridan, J Evans, M Jay, M Richards, J Montgomery, M Pellegrini), pp. 341-368. Oxford, UK: Oxbow Books.

[38] Evans JA, Chenery CA, Mee K, Marchant AP. 2023. Biosphere Isotope Domains GB (V2): Interactive Website. British Geological Survey. (Interactive Resource). (10.5285/2ce7fc22-1b6e-4979-968f-42058c0120fb)

[39] Pester M, Knorr KH, Friedrich MW, Wagner M, Loy A. 2012 Sulfate-reducing microorganisms in wetlands - fameless actors in carbon cycling and climate change. Front Microbiol. **28**, 72. (10.3389/fmicb.2012.00072) PMC3289269 22403575

[40] Newton J. 2021 An insect isoscape of UK and Ireland. Rapid Commun. Mass Spectrom. **35**, e9126. (10.1002/rcm.9126) 34008249

[41] Stack PE, Rock L. 2011 A *δ*^34^S isoscape of total sulphur in soils across Northern Ireland. Appl. Geochem. **26**, 1478-1487. (10.1016/J.APGEOCHEM.2011.05.021)

[42] Brunning R. 2009 Muchelney, Eastmoor Lane. In Somerset archaeology, vol. 153 (eds N Payne, CJ Webster), pp. 212-213. Taunton, UK: Somerset Archaeology and Natural History.

[43] Higbee L. 2011 Muchelney: The animal bone. Unpublished report, Wessex Archaeology. See https://www.wessexarch.co.uk/archaeological-services/publication.

[44] Brunning R. 2013 Burtle, Burtle Priory. In Somerset archaeology 2013 (ed. CJ Webster), pp. 144-165. Taunton, UK: Somerset Archaeology and Natural History.

[45] Higbee L. 2014 Animal bone. In Wessex Archaeology, Burtle Priory, Somerset: Specialist finds reports. Unpublished report, Wessex Archaeology. See https://wessexarch.co.uk/archaeological-services/publication.

[46] Brunning R. 2016 Glastonbury, Beckery Chapel. In Somerset archaeology 2016 (ed. CJ Webster), pp. 183-203. Taunton, UK: Somerset Archaeology and Natural History.

[47] Higbee L. 2016 Beckery Chapel: Animal Bone. Unpublished report, Wessex Archaeology. See https://wessexarch.co.uk/archaeological-services/publication.

[48] Patten R. 2009 Excavations at Eye Quarry. The Southern Extension: Phases 1, 2 and 3. Cambridge Archaeological Unit Report No. 869. Cambridge, UK: Cambridge Archaeology Unit.

[49] Evans C, Tabor J, Vander Linden M. 2016 Twice-crossed river: prehistoric and palaeoenvironmental investigations at Barleycroft farm/Over, Cambridgeshire. Cambridge, Cambridgeshire, UK: MacDonald Institute for Archaeological Research, University of Cambridge.

[50] Hall C. 2000 The excavation of terminal Bronze Age & medieval settlement remains at Baston Quarry (no.2), Langtoft, Lincolnshire. Phase IV, Area A. Cambridge Archaeological Unit Report No. 288. Cambridge, UK: Cambridge Archaeological Unit.

[51] DeNiro MJ. 1985 Postmortem preservation and alteration of *in vivo* bone collagen isotope ratios in relation to palaeodietary reconstruction. Nature **317**, 806-809.

[52] Nehlich O, Richards MP. 2009 Establishing collagen quality criteria for sulphur isotope analysis of archaeological bone collagen. Archaeol. Anthropol. Sci. **1**, 59-75.

[53] Chenery C. 2018 Biosphere Isotope Domain Map GB (V1): sulphur isotope data. British Geological Survey. See 10.5285/d023376c-08e3-451b-9d57-de13f14726bd.

[54] Evans JA, Chenery CA, Mee K, Cartwright CE, Lee KA, Marchant AP, Hannaford L. 2018 Biosphere Isotope Domains GB (V1): Interactive Website. British Geological Survey. (Interactive Resource). See https://www.bgs.ac.uk/dataset/biosphere-isotope-domains-gb/.

[55] Keeling CD. 1979 The Suess effect: 13carbon-14carbon interrelations. Environ. Int. **2**, 229-300. (10.1016/0160-4120(79)90005-9)

[56] Jauvion C, Bernard S, Gueriau P, Mocuta C, Pont S. 2020 Exceptional preservation requires fast biodegradation: the case study of thylacocephalan specimens from La Voulte-sur-Rhône (Callovian, Jurassic, France). Palaeontology **63**, 395-413. (10.1111/pala.12456)

[57] Piper DZ, Calvert SE. 2009 A marine biogeochemical perspective on black shale deposition. Earth Sci. Rev. **95**, 63-96. (10.1016/j.earscirev.2009.03.001)

[58] Chu TH, Bonnell LM, Anderson TF. 1993 Speciation and isotopic composition of sulfur in the Oxford Clay Formation (Jurassic, U.K.). Chem. Geol. **107**, 443-445.

[59] Bottrell S, Raiswell R. 1989 Primary versus diagenetic origin of Blue Lias rhythms (Dorset, UK): evidence from sulphur geochemistry. Terra Nova **1**, 451-456.

[60] Rippon S. 2004 Making the most of a bad situation? Glastonbury Abbey, Meare, and the medieval exploitation of wetland resources in the Somerset Levels. Medieval Archaeol. **48**, 91-130. (10.1179/007660904225022816)

[61] Bryant EA, Haslett SK. 2002 Was the AD 1607 coastal flooding event in the Severn Estuary and Bristol Channel (UK) due to a tsunami? Archaeology Severn Estuary **13**, 163-167.

[62] Horsburgh K, Horritt M. 2006 The Bristol Channel floods of 1607– reconstruction and analysis. Weather **61**, 272-282. (10.1256/wea.133.05)

[63] Lamb AL, Chenery CA, Madgwick R, Evans JA. 2023 Wet feet: developing sulfur isotope provenance methods to identify wetland inhabitants. Figshare. (10.6084/m9.figshare.c.6858126) PMC10565411 37830031

